# Facing Real-World Challenges of Immunogenicity in Pediatric Inflammatory Bowel Disease

**DOI:** 10.3389/fimmu.2020.01148

**Published:** 2020-06-09

**Authors:** Kyle Gress, Julie A. Bass, Ryan S. Funk, Ryan P. Morrow, Rachel Hasenkamp, Valentina Shakhnovich

**Affiliations:** ^1^Georgetown University School of Medicine, Washington, DC, United States; ^2^Department of Pediatrics, University of Missouri-Kansas City School of Medicine, Kansas City, MO, United States; ^3^Children's Mercy Kansas City, Kansas City, MO, United States; ^4^Department of Pharmacy Practice, University of Kansas Medical Center, Kansas City, KS, United States

**Keywords:** immunogenicity, children, inflammatory bowel disease, TNF-α, infliximab, adalimumab, biologics, anti-drug antibodies

## Introduction

The advent of biological therapies drastically altered the landscape of inflammatory bowel disease (IBD) treatment, making long-term steroid-free remission possible for thousands of patients living with this chronic inflammatory condition that compromises the integrity of the gastrointestinal mucosa. Unfortunately, up to 65% of patients with IBD develop anti-drug antibodies to biologics (1). This is especially problematic for pediatrics, where treatment options are substantially more limited than for adult patients. Currently, only two biologics have approval from the United States (U.S.) Food and Drug Administration (FDA) for pediatric indications in IBD, anti-TNF-α agents infliximab (IFX), and adalimumab (ADM). The fear of losing these two agents to immunogenicity is very real for the providers and the families of the ~70,000 children affected by IBD in the U.S. (2).

## General Factors Contributing to Immunogenicity

Immunogenicity, or the development of anti-drug antibodies (ADAs), is a major contributor to loss of treatment response to anti-TNF-α agents. Multiple factors play a role in ADA development and are frequently divided into drug properties, drug pharmacokinetics, and individual patient characteristics.

Drug properties, including compound structure and derivation, formulation and route of administration, play a significant role in immunogenicity. Briefly, compounds that are non-glycosylated, non-pegylated and/or non-human derived (i.e., chimeric) are more likely to elicit an immune response and be recognized as “non-self” by a patient's immune system, triggering ADA formation (3). Similarly, ADA formation is more likely to occur when drug concentrations are low (e.g., trough before the next dose) and the addition of new drug may challenge the host immune system to recognize the drug as “foreign.” Known factors associated with low trough concentrations are low drug dose, infrequent dosing, and accelerated drug clearance, observed when inflammatory burden is high and serum albumin (a marker of reduced Fc Receptor-mediated protein recycling) is low (4, 5). Lastly, compared to less concentrated intravenous formulations administered directly into the intravascular space, biologics administered subcutaneously are prone to protein aggregation and more likely to predispose to ADA development due to prolonged contact time with cutaneous and subcutaneous immune cells (3, 6).

Interestingly, when comparing the subcutaneously administered humanized biologic, ADM, to the intravenously administered chimeric biologic, IFX, data from multiple clinical trials, early on, demonstrated similar degree of immunogenicity for these two anti-TNF-α agents in patients with IBD (5). However, a more recent review of the IBD literature suggests that immunogenicity is up to two-fold greater for IFX than ADM (1), mirroring our clinical experience with these agents. Importantly, compared to all other autoimmune, inflammatory conditions treated with anti-TNF-α agents (e.g., rheumatoid arthritis, psoriasis, etc.), immunogenicity to IFX is highest in IBD (7).

## Immunogenicity Factors Unique to IBD

Mucosal erosion of the gastrointestinal epithelium, characteristic of IBD, predisposes patients with IBD to protein losing enteropathy, a condition that results in significant, abnormal protein losses in the stool, including the loss of protein-based therapies (8). In patients with IBD, increased stool losses of IFX have been linked to lower circulating IFX drug concentrations and increased propensity for IFX ADA development, with subsequent therapeutic failure and the need for total parenteral nutrition dependence, surgical intervention, and permanent bowel resection (9). Thus, ADA development in IBD goes beyond clinical manifestations of infusion reaction, serum sickness, and decreased drug efficacy (10), and poses a serious threat to patient morbidity and mortality.

With loss of treatment response estimated as 13% per patient-year of IFX therapy (11), children, who inherently have longer treatment duration than patients with adult-onset disease, are at greatest risk for losing biological treatment options, especially when those options are already limited to anti-TNF-α agents.

## Immunogenicity in Children

Although, generally, the pharmacokinetics of anti-TNF-α agents are believed to be similar between adults and children (12–14), data specifically comparing immunogenicity in adult vs. pediatric patients are lacking, and are confounded by the use of different ADA assays across studies. Nevertheless, it is well-established that therapeutic immunogenicity susceptibility varies with age, with highest susceptibility observed in the elderly and the young (3). Anecdotally, younger children also appear to clear anti-TNF-α agents faster, requiring higher, more frequent drug dosing in order to avoid immunogenicity and maintain treatment response (15). One proposed mechanism for this increased drug clearance is age-related differences in metabolic rate (16, 17), which, on a kilocalorie-per-kilogram basis, is highest during childhood.

Unlike conventional low-molecular weight drugs (i.e., ≤ 1 kDa), systemic clearance of protein-based therapies depends on proteolytic degradation (i.e., catabolism), determined in large part by metabolic rate, which depends on age, size and body mass composition (18). Highest proteolytic catabolism is expected in young, small, thin children—the typical clinical phenotype of pediatric patients with IBD, whose growth is frequently stunted by disease (19). Indeed, it has been suggested that close therapeutic drug monitoring and ADA surveillance for biologics may be most important for those pediatric patients who weigh less (4).

## Therapeutic Drug Monitoring

In our opinion, aside from medication adherence, therapeutic drug monitoring (TDM) is the single, most critical step for both preventing and overcoming immunogenicity in clinical practice. Clinical trial results from as early as 2014, demonstrate the cost-effectiveness of TDM for anti-TNF-α agents (20) and recent reports in pediatrics provide evidence that close TDM can help not only detect, but also reverse immunogenicity, with appropriate TDM-based dose adjustments (15).

At our center, between 2015 and 2018, TDM was performed 677 times for the ~350 children receiving anti-TNF-α therapy for IBD (21). Forty-five children (13%) were identified to have ADAs, and anti-TNF-α therapy was salvaged in 33% (14 IFX, 1 ADM) by increasing drug dose, shortening the dosing interval, and/or adding an immunomodulator to clear ADAs, as described by others (22). The other 30 children required prior authorization and appeals to third-party payers (e.g., letters of medical necessity, peer-to-peer communications) to secure off-label treatment with agents other than anti-TNF-α (e.g., ustekinumab, vedolizumab). To date, we have not detected immunogenicity with these newer agents.

## ADA Detection Platforms

In practice, the issue of testing for immunogenicity as part of proactive TDM is complicated by the availability of multiple ADA detection platforms. The intricacies of different ADA assay types are often unfamiliar to medical providers, with assay selection sometimes driven by third-party payer preference, or payment-support programs available to patients, especially if paying out of pocket. For example, based on financial considerations, providers at our institution alternate ordering ligand binding immunoassays, homogenous mobility shift and gene-reporter assays for therapeutic drug monitoring of biologics.

Of the currently available assays, providers are likely most familiar with ligand binding immunoassays (i.e., EIA, ELISA, ECLIA); however, there have been a number of novel ADA detection platforms developed, including homogenous mobility shift assays, gene-reporter assays, surface plasmon resonance, bio-layer interferometry, and mass spectrometry-based approaches (23). Although the overall correlation across these assays is acceptable (24), a major challenge in interpreting assay comparability is the use of different analytical standards and outcome measures that make interpretation of each assay highly dependent on the individual assay utilized (25). A major source for the observed variation amongst assays is the positive controls used in the assay, which commonly represent polyclonal ADAs developed through immunization of different animal species with the biological agent. The lack of uniform controls and reagents limits the comparability of results across assays and reveals the need for the development of ADA “standards” for the calibration and comparison of the various assays. This issue is perhaps best illustrated by comparing immunogenicity data from biosimilar development programs for IFX, which, overall, have failed to demonstrate a significant difference in the incidence of immunogenicity between the biosimilar and the innovator product. However, if one reviews the actual reported ADA incidence from study to study, it varies from 26 to 60%, based on the immunogenicity assay used (26–29). One consequence of the deficiency in uniform assay standards is dissemination of assay-specific treatment recommendations (25), which are not always clinically useful or applicable.

An added challenge in immunogenicity interpretation is the issue of drug tolerance, or unreliable ADA detection when free drug is present in the blood sample being tested. Some ADA platforms have improved the drug tolerance of immunoassays by adding an acid dissociation step to liberate ADAs bound to drug, while others have not, making comparisons across assays difficult.

Another important consideration in evaluating the clinical implications of immunogenicity is the differentiation of neutralizing vs. non-neutralizing ADAs. The differentiation is based on the ability of an ADA to directly interfere with the binding site of the biological agent, preventing its intended function at the drug target and, effectively, neutralizing drug activity/efficacy. Although neutralizing ADAs are believed to have the most clinical relevance, as they affect drug pharmacodynamics, non-neutralizing ADAs may also have significant impact on pharmacodynamics through pharmacokinetic alterations that result in lower drug exposure, secondary to reduced drug bioavailability and/or enhanced drug clearance mediated by ADA binding (30). To our knowledge, differentiation of ADA types is not routinely communicated in clinical immunogenicity reports. Although this information may be of benefit for clinical decision making, it could potentially drive up assay costs as three separate, validated methods would need to be applied in a tiered fashion to provide meaningful drug concentration, neutralizing and non-neutralizing ADA data.

Lastly, although the turn-around time for immunogenicity reports has improved greatly, results may still take up to 5 business days and point-of-care platforms, though available (31), are not yet integrated into routine clinical care.

## Discussion

In summary, despite the outlined evidence that pediatric patients with IBD are at increased risk for immunogenicity, and the knowledge that approved biologic treatments for children are limited to anti-TNF-α, clinicians face many challenges in implementing judicious, proactive therapeutic drug monitoring to detect immunogenicity in every-day IBD practice. A common barrier to implementing TDM is third-party payers denials to cover testing (21), despite the growing number of publications describing the clinical benefit and cost-effectiveness of TDM, specifically for anti-TNF-α therapy in IBD. (20, 32, 33) In practice, assay selection for TDM is often driven by financial considerations, and multiple ADA platforms may be used interchangeably for a given patient, confounding both the reliability and interpretability of test results.

In our opinion, uniformly validated ADA detection methods (e.g., standard reagents and positive controls), and provider education regarding limitations of different ADA assay types, could facilitate comparability of results across the different ADA platforms available. While, language regarding treat-to-target approaches and routine ADA assessment in the drug label, along with integration of point-of-care assays into clinical practice, could facilitate accessibility and affordability of TDM and ADA surveillance for patients and providers, preserving drug efficacy over time.

## Author Contributions

VS conceived the original idea for manuscript. JB, RF, RH, and VS provided expert opinion. KG, JB, RF, RM, RH, and VS conducted pertinent literature review, wrote and edited the manuscript, reviewed, and approved the final manuscript.

## Conflict of Interest

The authors declare that the research was conducted in the absence of any commercial or financial relationships that could be construed as a potential conflict of interest. The handling Editor declared a past co-authorship with one of the authors VS.
